# Infection and the evaluation of biomarkers in obstetrics and gynecology patients with infectious disease: a retrospective observational study from clinical pharmacists’ consultation experience

**DOI:** 10.1186/s40001-022-00850-5

**Published:** 2022-11-03

**Authors:** Jing Jin, Xiucong Fan, Xiaohui Dong, Xiaobo Zhai, Yabin Ma, Jing Tang

**Affiliations:** 1 Department of Pharmacy, The Obstetrics and Gynecology Hospital of Fudan University, 128 Shenyang Road, Yangpu District, Shanghai, 200090 China; 2 Department of Pharmacy, Shanghai East Hospital, Tongji University School of Medicine, 1800 Yuntai Road, Pudong District, Shanghai, 200120 China; 3 Department of Hospital Infection Management, Shanghai East Hospital, Tongji University School of Medicine, 1800 Yuntai Road, Pudong District, Shanghai, 200120 China

**Keywords:** Gynecological infection, Biomarkers, Pathogenic bacteria, Pharmacists’ consultations

## Abstract

**Background:**

The use of empirical anti-infective medication calls for the identification of common pathogens and accurate infectious biomarkers. However, clinical pharmacists’ anti-infective experience in the field of obstetrics and gynaecology is rare in the literature. This study aimed to retrospectively analyze the correlation between the anti-infective effectiveness after 7 days of antibiotic treatment and infectious biomarkers, according to clinical pharmacists’ consultation cases of gynecological and obstetric infections.

**Methods:**

In this retrospective study, clinical pharmacists’ anti-infective consultation experiences applied by physicians from January 1, 2018, to December 31, 2020, were included. The exclusion criteria were as follows: (1) the patient died or left the hospital before undergoing an effectiveness evaluation after the consultation; (2) treatment was discontinued due to adverse reactions related to antibiotics; (3) the patient did not undergo an effectiveness evaluation within 3 or 7 days after application of the clinical pharmacist’s treatment plan; and (4) the physician did not adopt the clinical pharmacist’s suggestions. The registered information included patient characteristics, pathological bacteria, anti-infective medication and changes in infection indices before and after treatment. Statistical analysis of temperature, white blood cells (WBCs), C-reactive protein (CRP), procalcitonin (PCT) and WBCs in urine after 3 days and 7 days of anti-infective treatment, compared with before anti-infective treatment, was performed by the chi-square test. A t test was conducted to further study WBC count and CRP. A receiver operating characteristic curve verified the sensitivity and specificity of WBC count, CRP and PCT.

**Results:**

A total of 265 cases were included. The CRP levels of patients 3 d and 7 d after antibiotic treatment were significantly lower than before antibiotic treatment (*P* < 0.05, *P* < 0.01), while the WBC count showed a downward trend after 3 days and a significant decrease after 7 days (*P* < 0.01). The areas under the curve (AUCs) for prognosis on the 7th day for WBC count, CRP and PCT were 0.90, 0.75 and 0.522, respectively. The AUC for WBC count combined with CRP was 0.90, which was higher than that for the biomarkers tested separately, especially compared to PCT. The most common gynecological infections were surgical site infection (SSI), urinary tract infection and fever of unknown origin, and the most common pathogens were *E. coli* and *E. faecalis* in Gram-negative and Gram-positive samples, respectively. Pharmacists’ recommended treatment plans included carbapenems and β-lactam antibiotics.

**Conclusions:**

Our dual-center study indicates that the combination of WBC count and CRP can improve diagnostic accuracy and treatment efficiency, and PCT alone is insensitive to gynecological infections, according to clinical pharmacists’ experience.

## Background

In October 2015, the World Health Organization (WHO) launched the Global Antimicrobial Resistance and Use Surveillance System (GLASS), in which more than 50 countries participate. GLASS data showed that antibiotic resistance is widespread among 500,000 people suspected of bacterial infection in these countries, which proved that bacterial infection had become a major challenge for the global health system and posed an increasing threat to human health. However, on a global scale, few studies have focused on anti-infective treatment in the specialties of obstetrics and gynaecology, especially the pathogens and biomarkers that indicate infections.

In China, due to the special rectification activities carried out nationwide, clinical pharmacists have actively participated in the rational use of antibiotics. In 2011, the Administration of Pharmaceutical Affairs in Medical Institutions launched regulations to promote the transformation of pharmacists’ roles from dispensing drugs to providing clinical pharmaceutical services [1], and the Antimicrobial Stewardship Program launched a pharmacist-led management method to strengthen clinical pharmacists’ status in the treatment of infectious diseases. With the strong support of relevant regulations, the Chinese Hospital Association has been committed to the training of clinical pharmacists and established a complete and effective training system for a series of diseases, including infectious diseases [2]. Therefore, clinical pharmacists with at least a master's degree in pharmacy can carry out clinical pharmacist consultations after systematic training in drugs for clinical diseases and successfully pass the examination. Zhang et al. [3] found that the effect of clinical pharmacists’ consultations on infectious disease treatment is vital and that the value of clinical pharmacists in this area should be recognized by decision-makers and hospital managers. Fan et al. [4] pointed out that on the basis of anti-infective consultations, clinical pharmacists integrated multidisciplinary individualized medication recommendations and improved patient outcomes. Based on Chinese clinical pharmacists’ involvement, their anti-infective consultations gradually developed as a body of knowledge composed of accumulated anti-infective experiences. However, according to an analysis of 1651 cases in tertiary hospitals in 10 provinces, the development of pharmaceutical consultations in obstetrics and gynaecology is far behind that in other departments [5]. Only Wang et al. [6] studied telephone consultations conducted by clinical pharmacists in obstetrics and gynaecology hospitals. However, the content of the telephone consultations was quite different from that of clinical pharmacists’ anti-infective consultations that are applied by physicians, which is of little relevance for infectious diseases.

It is well-known that the formulation of an anti-infective plan is based on the types of pathogens and infection sites. In addition, the biomarkers PCT, WBC count and CRP, which indicate the severity, prognosis and outcomes of infections, are of great use in clinical treatment. PCT, as an index with high sensitivity and specificity for the detection of infectious diseases, has proven its value for sepsis and respiratory system and bloodstream infections [7–10]. CRP and WBC count are commonly used as biomarkers in clinics. WBC-level and CRP-level kinetics have become focal points of research endeavours for the identification and assessment of inflammation prognoses [11, 12]. Their sensitivity and specificity were different for different bacterial infections. There are few studies on gynecological infections. Fernández et al. [13] pointed out that the positivity rate and value of CRP in acute and chronic pelvic inflammation were substantially higher than those in other cases, whereas it was low in nongonococcal urethritis and cervicitis, and the differential diagnosis was difficult. Nisenblat et al. [14] investigated the clinical value of the detection of CRP and WBC count in the differential diagnosis of gynecological infections, with highly accurate results for the combined detection of CRP and WBC count, which can be used as a routine diagnosis and treatment method to assist clinical screening and early guidance. As a result, clinical pharmacists who are engaged in anti-infective treatment can share their experiences in terms of the biomarkers above.

This study aims to summarize common gynecological pathogens, integrate targeted medication plans, and analyze the effectiveness of biomarkers that indicate infections, including PCT, WBC count and CRP, alone or in combination to promote the rational use of antibiotics in the field of obstetrics and gynecology based on clinical pharmacists’ experience.

## Methods

We followed the STROBE Statement for cohort studies to conduct this study.

### Study design and setting

This study was conducted at Fudan University Affiliated Obstetrics and Gynecology Hospital and Tongji University Affiliated East Hospital. In accordance with the ethical standards of the Helsinki Declaration, the Ethics Committee of the Obstetrics and Gynecology Hospital affiliated with Fudan University approved this study design with approval number 2021-115. This was a retrospective study with an informed consent exemption.

### Subject

In this retrospective study, clinical pharmacists’ anti-infective consultations applied by physicians from January 1, 2018, to December 31, 2020, were included. The exclusion criteria were as follows: (1) the patient died or left the hospital before undergoing an effectiveness evaluation after the consultation; (2) treatment was discontinued due to adverse reactions related to antibiotics; (3) the patient did not undergo an effectiveness evaluation within 3 or 7 days after application of the clinical pharmacist’s treatment plan; and (4) the physician did not adopt the clinical pharmacist’s suggestions.

### Consultation intervention

The clinical pharmacists’ anti-infective consultation intervention was described as follows: (1) Physicians requested a clinical pharmacist consultation on anti-infective treatment through the workstation. (2) After receiving consultation requests from physicians, clinical pharmacists evaluated the patients’ infections and made recommendations based on the purpose for the consultations (i.e., empirical application, drug adjustment and dosage adjustment). (3) Physicians made the final decision on whether to apply the recommendations.

### Variables and outcomes

This study was conducted on the basis of physicians who adopted the consultation recommendations and applied the medication plans provided by clinical pharmacists. The types of consultations proposed by physicians can be divided into two types: general consultations and multidisciplinary difficult consultations. General consultation is defined as only the clinical pharmacist being invited to give recommendations for the purpose of treating the infection of a specific patient. Multidisciplinary difficult consultation means that for some patients who need multidisciplinary medical support, a clinical pharmacist is invited as part of the multidisciplinary medical team to give recommendations regarding the infection. ROC curves were used to estimate the sensitivity and specificity of the biomarkers in infected patients. The optimal threshold for ROC curves was defined as the value maximizing the sum of the sensitivity and specificity. The 7-day prognosis was defined as the restoration of ear temperature and inflammatory biomarkers to normal, while pathogen cultures became negative at day 7. The normal ranges of ear temperature, WBC count, CRP, PCT and WBCs in urine were defined as 36.5–37.5 °C, 3.5–9.5 × 10^9^/L, 0–10 mg/L, 0–0.5 ng/ml and 0–40/μl, respectively. We selected alanine aminotransferase (ALT) and glutamic oxaloacetic aminotransferase (AST) as the judging indicators of liver function and serum creatinine (sCr) as the judging indicator of kidney function, with corresponding normal ranges of 7–40 units/L, 13–35 units/L, and 44–97 µmol, respectively.

The primary endpoints included (1) the improvement in infection indices (WBC count, CRP, and PCT) after antibiotic use and (2) the increased sensitivity and specificity of WBC count, CRP, and PCT, alone and in combination, after 7 days of antibiotic treatment. The secondary endpoints included (1) infection diagnoses of gynecological and obstetric consultation patients; (2) types of specimens for examinations; and (3) distribution of pathogenic bacteria and the corresponding anti-infective treatment plan in the consultations.

### Data collection

The investigators built a registry database to record relevant data, including patient identity, primary disease and infection information, sample sources, composition of pathological bacteria, anti-infective medication treatment and changes in infection indices before and after treatment (temperature and biomarkers). The data above came from the inpatient workstation and Hospital Information System (HIS). To avoid mistakes during collection, the data were crosschecked by investigators.

To reduce the bias of the method, the outcome evaluator was not the data collector or the provider of the anti-infective consultations.

### Statistics

The data of 265 consultation cases were ultimately included after case selection. Statistical analysis was conducted using SPSS 25.0 software (SPSS Inc., Chicago, IL, USA) for Windows. Pareto diagrams were used to demonstrate the most common infection diagnoses and the types of specimens in this study. The distribution of bacteria and corresponding treatment plans applied in the consultations were presented using a Sankey diagram. Normal distribution of data was confirmed using the Kolmogorov‒Smirnov test (*P* > 0.05). When the data followed a normal distribution, means and standard deviations (SDs) or medians and interquartile ranges were used to present them. After analysis of variance, a t test was used to analyze the data. If continuous data were not normally distributed, they were presented as medians with interquartile ranges (IQRs) and were compared using the Mann‒Whitney U test. Frequency, rate, or constituent ratio were utilized to count data, which were then analyzed by a chi-square test. Receiver operating characteristic (ROC) curves were plotted to evaluate the sensitivity and specificity of WBC count and CRP after 7 days of antibiotic therapy. P values less than 0.05 were considered statistically significant.

## Results

**Fig. 1:**
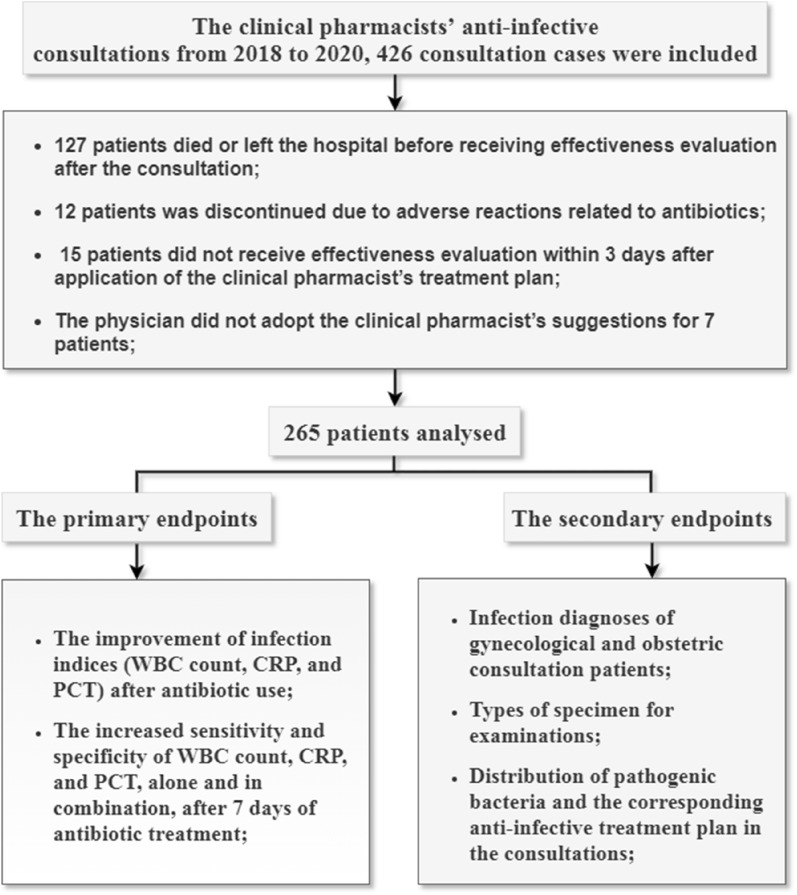
Flowchart of the case screening process and study design regarding the primary and secondary endpoints

A total of 426 cases of anti-infective consultations were collected and assessed for eligibility by clinical pharmacists. A total of 265 (62.2%) patients were finally enrolled and analysed. This study included two primary endpoints and three secondary endpoints, as detailed in Fig. 1.

As shown in Table 1, 265 cases were included in this study, among which 215 cases (81.13%) were from Fudan University Affiliated Obstetrics and Gynecology Hospital and 50 cases (18.87%) were from Tongji University Affiliated East Hospital. The characteristics of the consultations and proportions of patients with abnormal liver and renal function are given in Table 1.

**Table 1:** Characteristics of consultations and patients

	Consultation number (*n*)	Percentage
Department		
Gynecology Department	241	90.94
Obstetrics Department	24	9.06
Types of consultations		
General consultation	253	95.47
Multidisciplinary difficult consultation	8	3.02
Age group (y)	47.24 ± 13.69	
0–17	3	1.13
18–65	247	93.21
≥ 66	15	5.66
Kidney function		
Normal	241	90.94
Abnormal	24	9.06
Liver function		
Normal	243	91.70
Abnormal	22	8.30

**Fig. 2:**
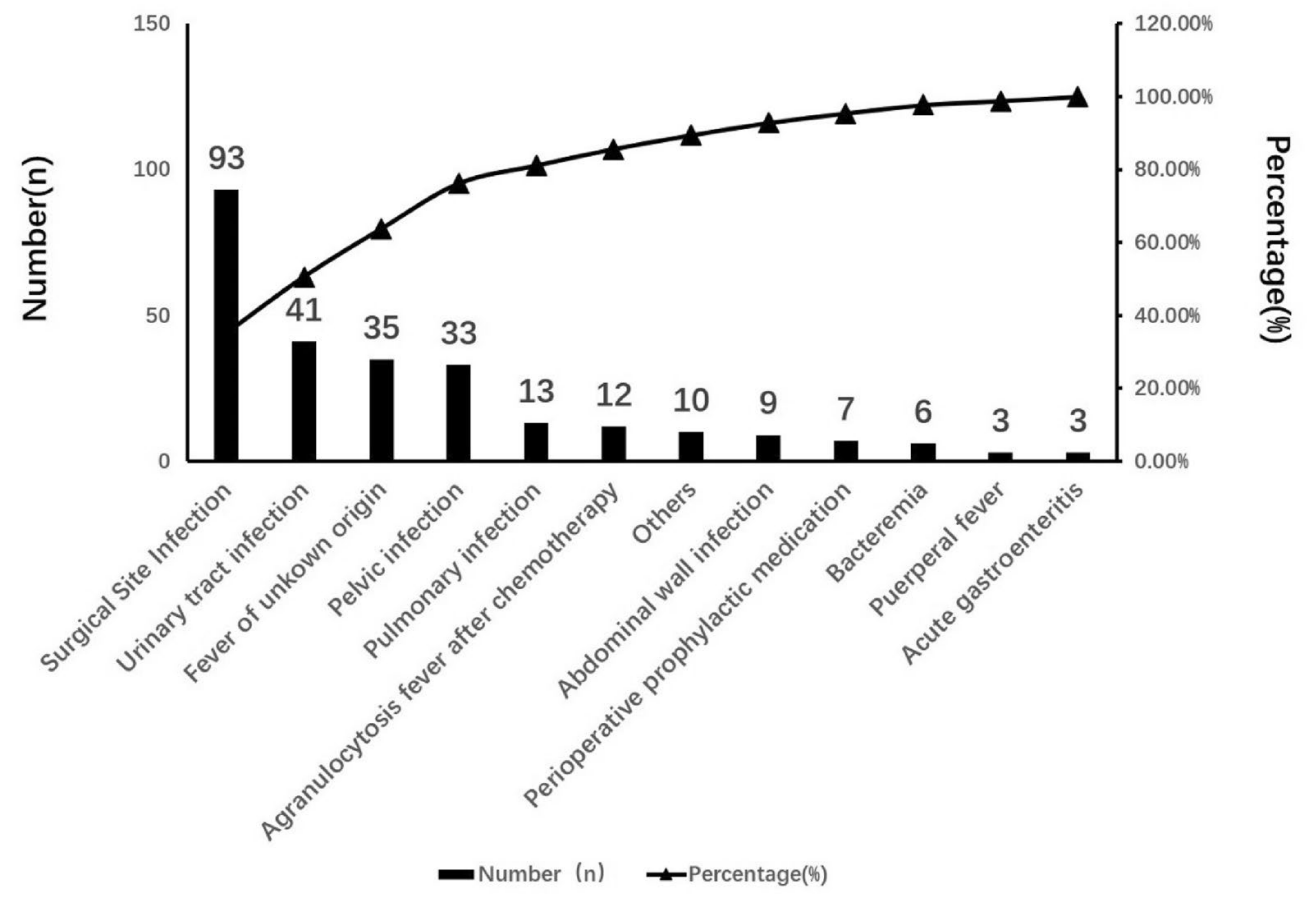
Infection-related diagnoses of the patients with infection in clinical pharmacists’ consultation cases

In terms of the infection diagnoses demonstrated in Fig. 2, the patients with SSI accounted for the largest part (93, 35.09%), followed by those with urinary tract infection (41, 15.47%), fever of unknown origin (35, 13.21%), pelvic infection (33, 12.45%) and pulmonary infection (13, 4.91%), which were closely related to anti-infective treatments.

**Fig. 3:**
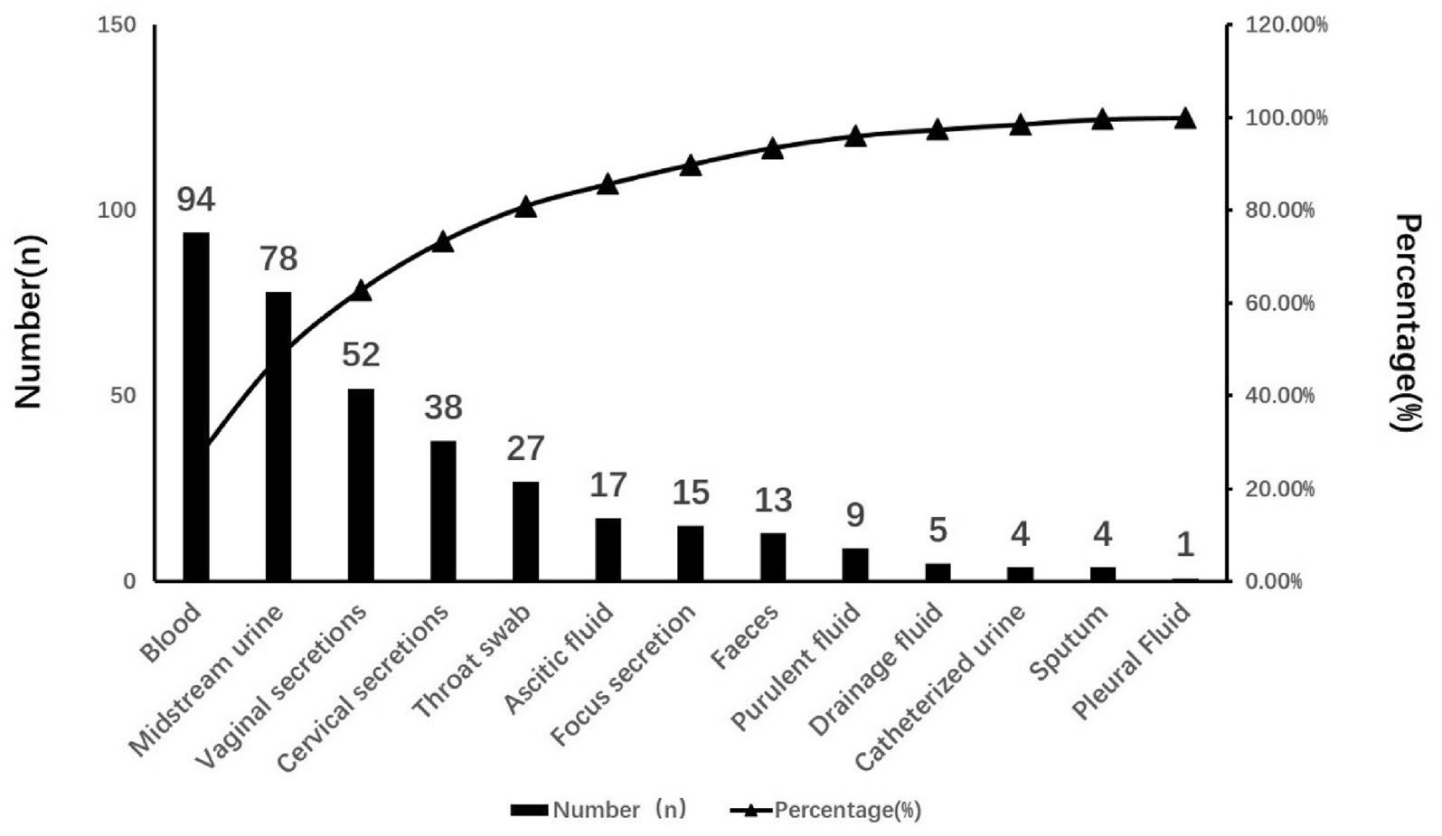
Types of samples collected from patients for pathogen detection

As shown in Fig. 3, the top 3 sample sources were blood (94, 35.47%), midstream urine (78, 29.43%) and vaginal secretions (52, 19.62%). There were 61 cases (23.02%) without samples. The top 4th and 5th sample types we collected for pathogen detection were cervical secretions and throat swabs. In this study, the most commonly used test for pathogen detection was the time-of-flight mass spectrometer test; next-generation sequencing and quantitative real-time PCR were among the most commonly used methods as well.

Regarding the composition of the pathogenic bacteria in Table 2, a total of 117 bacterial isolates were detected, where Gram-negative bacteria accounted for the highest proportion, followed by Gram-positive bacteria, fungi and atypical pathogens. In terms of Gram-negative bacteria, *E. coli*, *K. pneumonia* and *K. pneumonia* ranked in the top 3, while *E. faecalis*, *E. faecium* and *S. aureus* were the top 3 isolates among Gram-positive bacteria.

**Table 2:** Distribution of pathogenic bacteria

	Bacteria isolates	Number (*n*)	Percentage
Gram-negative	*E. coli*	36	13.58
	*K. pneumoniae*	9	3.40
	*P. mirabilis*	4	1.51
	*A. baumannii*	2	0.75
	*E. cloacae*	2	0.75
	Other Gram-negative bacteria (*M. morganii, P. morganii, S. marcescens*)	4	1.51
Gram-positive	*E. faecalis*	16	6.04
	*E. faecium*	13	4.91
	*S. aureus*	8	3.02
	*S. pneumoniae*	4	1.51
	*S. agalactiae*	2	0.75
	*S. epidermidis*	1	0.38
	Other Gram-positive bacteria (*S. dyslactiae, S. wallichii, S. ludens*)	3	1.13
Fungus	*C. albicans*	3	1.13
	*C. tropicalis*	2	0.75
	*C. glabrata*	1	0.38
	*C. lusitaniae*	1	0.38
	mould	1	0.38
Atypical pathogen	Ureaplasma urealyticum	3	1.13
	Mycoplasma hominis	2	0.75
Negative results		148	55.85
Total		265	100.00

**Fig. 4:**
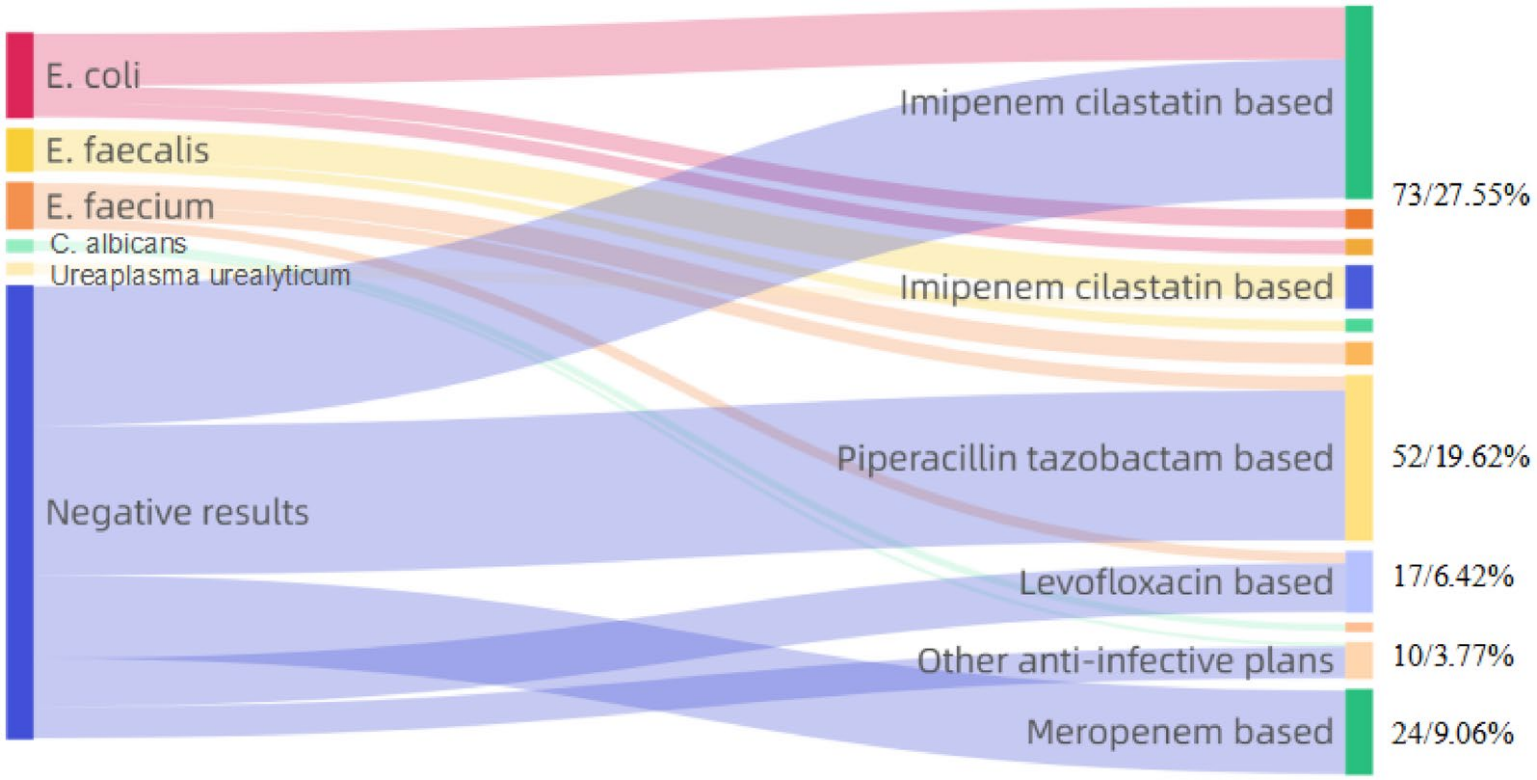
Top five pathogens detected in the patients and the corresponding treatment regimens Notes: Imipenem–cilastatin-based: Imipenem–cilastatin ± amikacin/azithromycin/fluconazole/fosfomycin/vancomycin. Piperacillin tazobactam based: Piperacillin tazobactam ± amikacin/ornidazole/fosfomycin/vancomycin/metronidazole/cefoxitin; Levofloxacin based: Levofloxacin ± amikacin/ornidazole/fosfomycin. Meropenem based: Meropenem ± fluconazole/fosfomycin/tigecycline/vancomycin/ornidazole

The choices of anti-infective treatment plans in consultations by clinical pharmacists are shown in Fig. 4. In this study, imipenem/cilastatin alone or in combination with other antibiotics was the first choice (73, 27.55%), with piperacillin/tazobactam (52, 19.62%) and meropenem (24, 9.06%) as the second and third choices, respectively.

The data in Table 3 show that the temperatures, WBC counts and CRP of patients 3 days and 7 days after antibiotic treatment were significantly higher than those before antibiotic treatment (*P* > 0.01). However, for PCT and WBCs in the urine of patients before antibiotic treatment compared to 3 d after antibiotic treatment, there were no significant differences (*P* > 0.05).

**Table 3:** Infection indices of the consultation patients

	Before antibiotic treatment	3d after antibiotic treatment (*n* = 265)	7d after antibiotic treatment(*n* = 265)
Temperature (℃, *n* = 265)			
Normal	55 (20.75%) 37.10 ± 0.24	208 (78.49%) 37.08 ± 0.23	250 (94.34%) 37.02 ± 0.21
Abnormal	210 (79.25%) 38.46 ± 0.63	57 (21.51%) 37.95 ± 0.56	15 (5.66%) 37.32 ± 0.79
χ2 value		176.682	293.672
*P* value		< 0.01	< 0.01
WBC (× 10^9^/L, *n* = 265)			
Normal	70 (26.42%) 6.68 ± 1.76	195 (62.26%) 5.70 ± 1.68	210 (79.25%) 5.94 ± 1.34
Abnormal	195 (73.58%) 13.06 ± 11.47	70 (37.74%) 16.65 ± 12.85	55 (20.75%) 15.55 ± 14.81
χ^2^ value		117.925	148.4
*P* value		< 0.01	< 0.01
CRP (mg/L, *n* = 265)			
Normal	42 (15.85%) 2.60 ± 1.78	90 (33.96%) 5.58 ± 4.77	229 (86.42%) 4.94 ± 2.25
Abnormal	223 (84.15%) 74.99 ± 44.92	175 (66.04%) 61.12 ± 44.21	36 (13.58%) 46.53 ± 28.65
χ^2^ value		23.243	264.052
*P* value		< 0.01	< 0.01
PCT ( ng/ml, *n* = 145)			
Normal	89 (61.38%) 0.15 ± 0.10	101 (26.79%) 0.15 ± 0.11	137 (94.48%) 0.14 ± 0.17
Abnormal	56 (38.62%) 23.65 ± 50.95	44 (16.60%) 46.44 ± 47.10	8 (5.52%) 82.55 ± 14.34
χ^2^ value		2.198	46.195
*P* value		0.138	< 0.01
WBC in urine (/μl, *n* = 59)			
Normal	26 (44.08%) 8.09 ± 9.89	36 (61.02%) 6.27 ± 9.86	44 (74.58%) 4.10 ± 7.29
Abnormal	33 (55.92%) 2817.86 ± 6385.10	23 (38.98%) 583.02 ± 789.09	15 (25.42%) 68.41 ± 41.18
χ^2^ value		3.399	11.379
*P* value		0.065	0.001

**Fig. 5:**
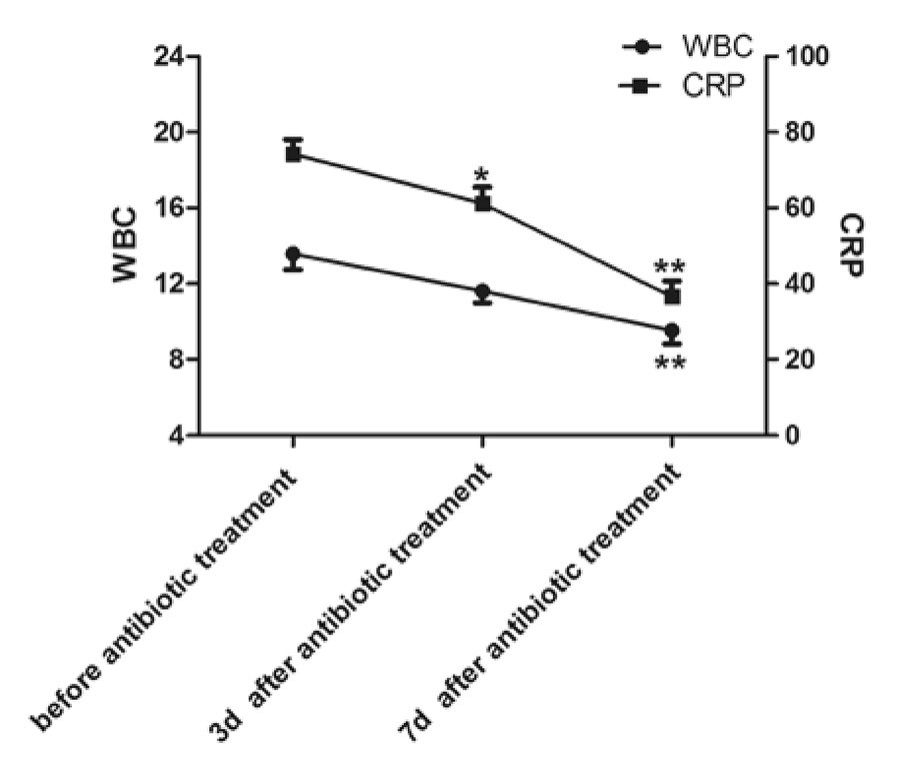
Antibiotic administration improves infection markers (WBC count and CRP)

After 3 and 7 days of antibiotic treatment, WBC count and CRP were measured and analyzed by paired t test (Fig. 5). In terms of WBC count, compared with before antibiotic treatment and 3 days after antibiotic therapy, there was no significant difference. Likewise, compared to before antibiotic treatment, the CRP levels declined significantly after 3 days of antibiotic therapy (*P* < 0.05) and 7 days of antibiotic therapy (*P* < 0.01).

**Fig. 6:**
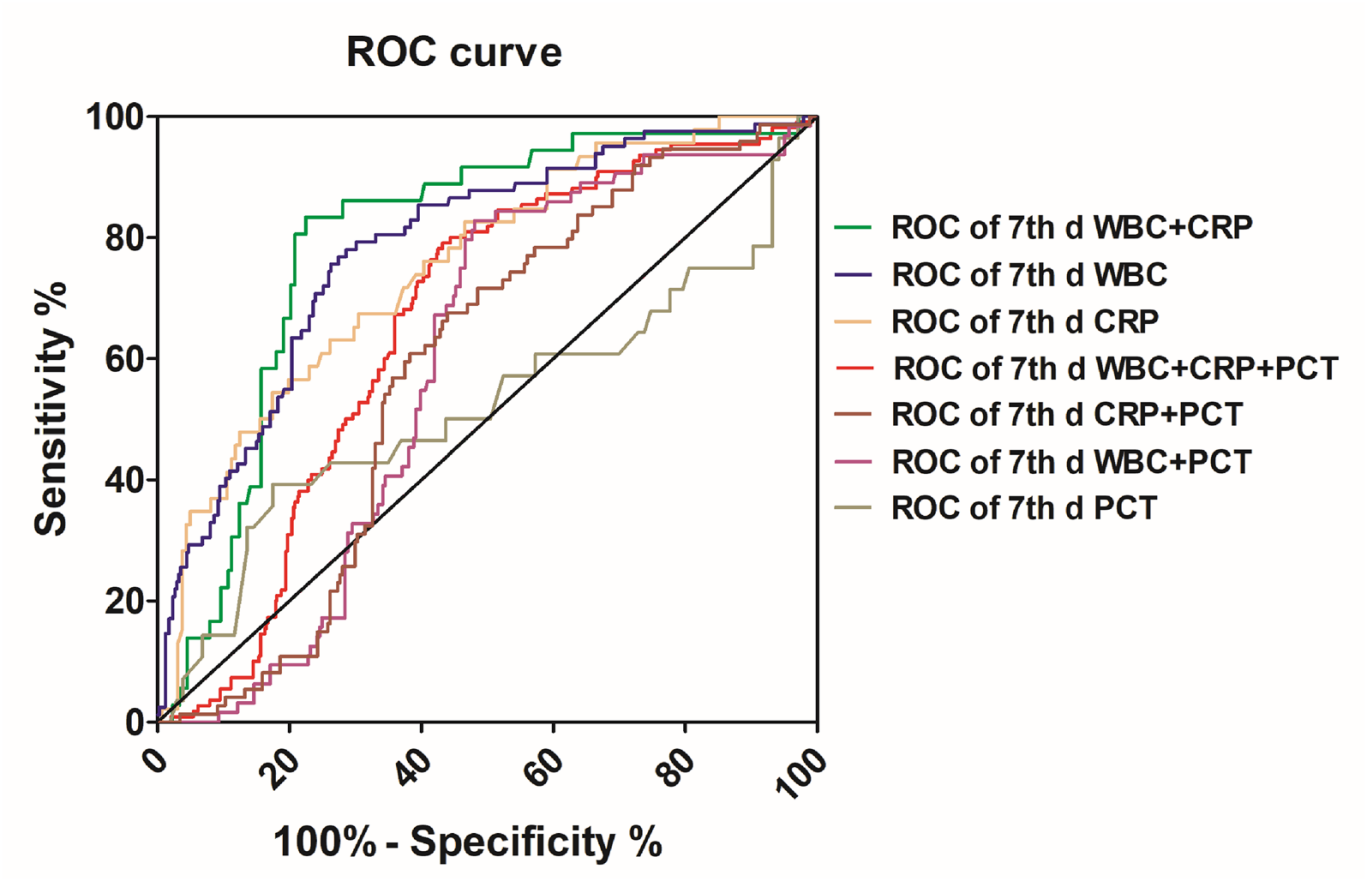
ROC curves for WBC count, CRP, and PCT, alone or in combination, after 7 days of antibiotic treatment. The curves show how sensitivity (true positive fraction) varies with specificity (false-positive fraction) when the diagnostic cutoff limit is varied

ROC curves for each studied biomarker and each combined biomarker are shown in Fig. 6. For a single biomarker, a WBC count cutoff value ≥ 9.5 × 10^9^/L showed a sensitivity of 100%, specificity of 86.41%, and highly significant accuracy (*P* < 0.001). The cutoff value of CRP ≥ 10 mg/L showed a sensitivity of 54.35%, specificity of 82.61%, and significant accuracy (*P* < 0.001). A cutoff value of PCT > 0.5 μg/L showed a sensitivity of 39.29%, specificity of 80.58%, and significant accuracy (*P* > 0.05). Furthermore, the peak areas under the ROC curves for WBC count, CRP and PCT for 7-day prognosis were 0.83 (95% CI 0.73–0.84), 0.75 (95% CI 0.67–0.83; *P* < 0.001), and 0.522 (95% CI 0.38–0.66; *P* > 0.05), respectively. For the combined biomarkers, the combination of WBC count and CRP for 7-day prognosis was better than only a single biomarker. The AUC for WBC levels combined with CRP was 0.90 (95% CI 0.84–0.95; *P* < 0.001). The AUC for the 7-day prognosis of CRP levels combined with PCT was 0.58 (95% CI 0.52–0.65), compared to an AUC of 0.59 (95% CI 0.52–0.65) for WBC count combined with PCT. The AUC for WBC count and CRP combined with PCT was 0.66 (95% CI 0.61–0.71; *P* < 0.001).

## Discussion

In clinical practice, to treat infectious diseases, doctors should focus on pathogens, biomarkers and medication-related problems, such as drug selection, administration, usage, and dosage of drugs. Only when all the ingredients above are taken into consideration can satisfactory therapeutic effects be achieved. In addition to referring to doctors’ clinical experience, clinical pharmacists, as the main clinical consultants of severe infectious patients in China, should summarize their experience to provide better medical and pharmaceutical services. To our knowledge, this is the first observational study to analyse common gynaecological pathogens, integrate targeted medication plans and screen the most valuable infectious indicators. Compared to previous studies, this retrospective study could offer insights from clinical pharmacists for doctors in the field of gynaecology and obstetrics when encountering patients with severe infections.

Previous studies have shown that the urinary tract is the main site of postoperative infection in patients who undergo gynecological surgery, followed by the respiratory system and incision [15]. SSI is the most common infection complication after gynecological surgery and mainly includes wound surface cellulitis, deep abscess, and pelvic or vaginal stump infection [16–18]. The results of this study showed that SSI was the most common infection, followed by urinary tract infection, fever of unknown origin, pelvic inflammatory disease and pulmonary infection. The site with the highest infection rate is not consistent with the literature; however, the types of diseases are similar, which may be attributed to the fact that consultation cases are mainly difficult cases identified by physicians, resulting in a low correlation with the actual incidence of infection. Mothes et al. [19] found that gynecological infections were mainly caused by Group B *Streptococcus*, *Escherichia coli*, *Klebsiella*, *Enterobacterales*, *Proteus* and other *Enterobacterales* bacteria, which was consistent with the results shown in this study. In this study, we found that the most recommended treatment plans included imipenem/cilastatin, piperacillin/tazobactam, and meropenem alone or in combination with other antibiotics. Clinical pharmacists selected the most proper medications mainly based on their antibacterial spectrum and toxicity for specific patients in some cases. Notably, according to the results in Table 3, the WBCs in the urine of patients diagnosed with urinary tract infection did not decrease significantly after 3 days of treatment, but the treatment effect was obvious after 7 days of treatment (*P* < 0.01), suggesting the importance of sufficient medication duration and measurement frequency.

CRP and WBC count are commonly used as biomarkers in clinics. They have become focal points of research endeavours for the identification and assessment of inflammation prognosis [11, 12]. WBC count is a viable biochemical marker of the severity of infection in patients. CRP values can increase more than 100 times over, which are more noteworthy than baseline values and show a functioning state of infection. Their sensitivity and specificity were different in different bacterial infections.

The PCT level marks the degree of inflammatory response activity and helps to clarify the colonization or invasion of a detected bacterial pathogen, which may contribute to choosing an empiric therapy [7, 20]. Previous studies have shown that PCT could distinguish Gram-negative from Gram-positive bacteria at a significantly higher level [21, 22]. However, some contradictory voices support that PCT levels are easily affected by a series of factors, including infection sites, anti-infective drugs and specific pathogens. Gram-negative bacteria are not the decisive factor [23–25]. In this study, we investigated the importance of PCT in clinical pharmacists’ treatment plans for gynecological infections. The results demonstrated that the effect of PCT level was not obvious after 7 days of antibiotic treatment, which was not consistent with the outcomes of WBC count or CRP (Table 3). This means that PCT did not show any value in assessing the prognosis of patients with gynecological infection. This may be attributed to the relatively small sample size of the study, which to a large extent depends on physicians’ clinical experience, lower accuracy of the test results and low capacity for rapid PCT assays, as was pointed out in many studies [25–27]. Moreover, our results showed that Gram-negative bacteria were the most common strains in consultations of obstetrics and gynecological infections. However, in terms of the infection sites, SSI ranked first (35.09%), resulting in the proportion of Gram-positive bacteria-induced infections being higher than that reported in the literature. This may also exert a negative impact on PCT levels in this study [28].

Rashwan et al. [29] revealed that PCT > CRP > WBC count in terms of sensitivity for bacterial infections and PCT > CRP > WBC in terms of specificity for common pathogenic bacteria. Li et al. [30] pointed out that the positivity rate and value of CRP in acute and chronic pelvic inflammation were significantly higher than those in other cases, whereas it was low in nongonococcal urethritis and cervicitis, and the differential diagnosis was difficult. Hasan et al. [31] investigated the clinical value of the measurement of CRP and WBC count in the differential diagnosis of gynecological infections, with highly accurate results for the combined detection of CRP and WBC count, which can be used as a routine diagnosis and treatment method to assist clinical screening and early guidance; this is consistent with our results. Our results showed that the combination of WBC count and CRP used for 7-day prognosis was superior to the use of a single biomarker and other combination regimens.

In this study, the outcomes in Fig. 5 show that both WBC count and CRP decreased on the 3rd and 7th days after anti-infective treatment, which was consistent with the results presented in Table 3 and demonstrates that the prognosis after anti-infective treatment is good. In Fig. 6, ROC curves were utilized to investigate the diagnostic efficiency of these biomarkers, including WBC count, CRP and PCT. Various examinations determined that the CRP test sensitivity ranged from 70% to 93%, specificity ranged from 41% to 98%, PPV ranged from 6% to 83%, and NPV ranged from 97% to 99% [28, 29]. Although the results appear to present normal sensitivity and specificity, as detailed by Çetinkaya et al., the increase in CRP levels is delayed during the first 24–48 h of disease, which negatively affects its specificity. In addition, the increase in the CRP level in noninfective cases severely affects its specificity. Therefore, based on our findings, we propose that to acquire a more accurate inflammation prognosis, CRP combined with WBC count could be utilized as an inflammation indicator. Studies have added that the advantages of WBC count over CRP are that its typical level increases mostly in bacterial infection, and its normal level is rapidly re-established after antibiotic treatment [31].

Since health administration authorities in China attached greater importance to the rational use of antibacterial drugs, which has been included in hospital management and performance evaluation indicators, clinical pharmacists have given more attention to the improvement in anti-infective consultations. In this study, the effectiveness rate of consultations (calculated by the number of patients whose biomarkers and symptoms returned to normal 7 days after treatment) was 94.34%. Clinical pharmacists are indispensable members of the treatment team. They can help physicians use antibiotics more safely and effectively, which could lead to better patient outcomes. Clinical pharmacists specializing in gynecological and obstetric systems should continuously improve their capabilities of dealing with problems such as individualized adjustment of medication plans based on blood drug concentrations and PK/PD characteristics. With more consultation requests and accumulated experience, clinical pharmacists could better serve patients and physicians troubled by infections.

## Conclusions

As clinical pharmacists focused on anti-infective treatment, we proposed that it is vital to pay attention to the combination of WBC count and CRP and the choice of medication between imipenem/cilastatin, piperacillin/tazobactam, and meropenem when physicians need anti-infective support. It is worth noting that the inspection value of PCT was limited in clinical pharmacist consultations on gynecological infections.

## Limitations

The limitations of this study are as follows: first, this is a retrospective dual-center study with a relatively small sample size. To minimize bias and provide a more reliable reference, prospective and multicentre studies with large sample sizes should be carried out. Second, these results should be interpreted with caution given the small sample size of PCT. Further research on biomarkers in obstetrics and gynecological infections is needed on the basis of increasing the test amount of inflammation indicators, which calls for clinical pharmacists and physicians to pay more attention to inflammation indicators when infection occurs.

## Data Availability

The data that support the findings of this study are available from the corresponding author upon reasonable request.
